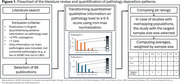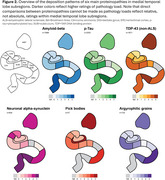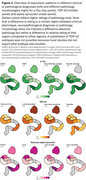# Deposition patterns of proteinopathies in medial temporal lobe subregions: implications for neuroimaging

**DOI:** 10.1002/alz70855_098998

**Published:** 2025-12-28

**Authors:** Laura E.M. Wisse, Maud M.A. Bouwman, Alex J. Wesseling, Anika Wuestefeld, Hannah Tucker, Daniel T Ohm, Paul A. Yushkevich, David A. Wolk, Laura E. Jonkman

**Affiliations:** ^1^ Lund University, Lund, Sweden; ^2^ Amsterdam UMC, location VUmc, Amsterdam, Netherlands; ^3^ Clinical Memory Research Unit, Department of Clinical Sciences Malmö, Lund University, Lund, Sweden; ^4^ Penn Frontotemporal Degeneration Center, Department of Neurology, Perelman School of Medicine, University of Pennsylvania, Philadelphia, PA, USA; ^5^ Perelman School of Medicine, University of Pennsylvania, Philadelphia, PA, USA; ^6^ Penn Memory Center, University of Pennsylvania, Philadelphia, PA, USA

## Abstract

**Background:**

The medial temporal lobe (MTL) is a hotspot for neurodegenerative pathologies and a key region for neuroimaging studies in dementia. A better understanding of proteinopathy deposition patterns would increase the interpretation of neuroimaging measures. We performed a literature review to quantitatively characterize deposition patterns in MTL subregions for six major proteinopathies targeting the MTL.

**Method:**

Figure 1 shows the flowchart for the literature review. In brief, to harmonize across publications, MTL pathology measures were transformed into a 0‐5 scale using min‐max normalization for each main proteinopathy (i.e., hyperphosphorylated tau (*p*‐tau), amyloid‐beta, alpha‐synuclein, TDP‐43, Pick bodies, and argyrophilic grains). Average pathology ratings were calculated per MTL subregion per proteinopathy, clinical‐pathological diagnosis, and pathology morphology (e.g., *p*‐tau threads vs tangles), weighted by the sample size of the publication.

**Result:**

Pathology load in MTL subregions was obtained from 99 publications. *p*‐tau deposition showed the highest load in the transentorhinal cortex (TERC), followed by entorhinal cortex (ERC), cornu ammonis (CA)1 and subiculum (Figure 2). TDP‐43 and argyrophilic grains showed a similar pattern, but with the highest load in the amygdala and ERC respectively. Pick bodies uniquely showed the highest load in the dentate gyrus and amygdala, followed by the ERC. Alpha‐synuclein and amyloid‐beta pathology showed highest load in the lateral MTL cortex, and amygdala for alpha‐synuclein. Different clinical phenotypes or neuropathological subtypes (Figure 3) for tau, TDP‐43, and alpha‐synuclein pathology showed similar distributions patterns with subtle differences. Only subiculum *p*‐tau relative rating was significantly higher in Alzheimer's disease neuropathologic change compared to primary age‐related tauopathy (weighted t‐test: t(3.41)=3.13; *p* = 0.04). Subtle differences were observed when comparing pathology morphology, with significantly higher relative ratings of *p*‐tau threads than neurofibrillary tangles in TERC (t(4)=‐3.40; *p* = 0.03), ERC (t(4)=‐3.39; *p* = 0.02) and CA1 (t(3.86)=‐3.10; *p* = 0.04), and significantly higher relative ratings of Lewy neurites than Lewy bodies in CA2 (t(7.75)=‐3.50; *p* = 0.008).

**Conclusion:**

While *p*‐tau, TDP‐43, and argyrophilic grain proteinopathies show similar deposition patterns across MTL subregions, other proteinopathies showed selective vulnerability to more unique MTL subregions. The identified deposition patterns may inform patterns of downstream neurodegeneration and guide the development of sensitive and specific imaging biomarkers across these heterogeneous proteinopathies.